# The effects of multicultural family support services on the longitudinal changes of acculturative stress, peer relations, and school adjustment

**DOI:** 10.3389/fpsyg.2023.1301294

**Published:** 2024-01-08

**Authors:** Hyun Seon Ahn, Jeesoo Lee, Yuanying Jin

**Affiliations:** ^1^Major of Education Methods, Graduate School of Education, Korea University, Seoul, Republic of Korea; ^2^Department of Teacher Training, Korea University, Seoul, Republic of Korea; ^3^Department of Education, College of Humanities, Sejong University, Seoul, Republic of Korea

**Keywords:** multicultural adolescents, longitudinal, acculturative stress, peer relations, school adjustment, MAPS 1, MAPS 2011

## Abstract

This study investigated the long-term trends of acculturative stress, peer relationship, and school adjustment among adolescents from multicultural families in South Korea, and examined the concurrent and lagged effects of multicultural family support services (MFSS) on the longitudinal changes in the variables of interest. Concurrent and lagged models as well as developmental trajectories via latent growth modeling (LGM) were employed on a sample of 1,443 middle school students (706 boys, 737 girls) from the Multicultural Youth Panel Survey (MAPS). The results revealed that peer relationships and school adjustment perceived by adolescents demonstrated consistent, gradual declines across 3-year time points while there was a lack of significant change in acculturative stress during the same period. The absence of significant change in acculturative stress could be attributed to floor effects, stemming from its initial low mean level. Nevertheless, the LGM modeling also exhibited significant concurrent and lagged effects of MFSS at the first and third time points of investigation. The intermittent significant effects of MFSS and the direction of its improvement were discussed. More educational interventions guiding students from multicultural families would be needed as they navigate the complexities of adolescence.

## Introduction

In today's globally interconnected milieu, the burgeoning multicultural population in South Korea underscores the need to understand the experiences in schools of adolescents with multicultural backgrounds. With the increasing number of such students (Kim, 2023a; Yoon, 2023), a recent proclamation by South Korea's Ministry of Gender Equity and Family has highlighted the government's commitment to inclusive education and emphasizes its dedicated support (Lee, 2022) for students from multicultural families, thereby promoting educational inclusivity and equity. Despite the long-standing emphasis on multicultural education by government programs in school settings, however, few studies have evaluated the effectiveness of these policy program initiatives.

In doing so, it is essential to incorporate key elements to understand their effects on students with multicultural backgrounds. Previous studies have shown that acculturative stress, a critical component of multicultural adaptation, profoundly influences the experiences of adolescents with multicultural backgrounds, specifically, in terms of its wide-ranging impacts on their academic performance (Albeg and Castro-Olivo, 2014), psychological wellbeing (Bae, 2019), school adjustment (Lim, 2021) and social interactions (Crockett et al., 2007). Moreover, research has revealed that multicultural students face a variety of challenges ranging from language and social relationships with teachers and peers to understanding of and adjustment to school environments (Lee and Song, 2011). Alongside these challenges, Choi (1997) has recommended that a support program is needed to promote intercultural and interpersonal relationship in helping immigrant students adjust. However, while considerable research has examined the structural relationships between acculturative stress, peer relations, school adjustment, the influence of government support policies on students with multicultural backgrounds remains less explored. Additionally, there is a notable gap in the literature in regard to examining the longitudinal trajectories of acculturative stress, peer relations, and school adjustment.

This study, therefore, has endeavored to shed light on how multicultural students' experiences of multicultural family support services influence their perceptions of acculturative stress, peer relations, and school adjustment, as well as how these perceptions change over time. In simultaneously examining the influence of multicultural support services on these longitudinal dynamics and evaluating the effects of government programs on the development of acculturative stress, peer relationship, and school adjustment, this research could potentially provide improved suggestions for enhancing the effectiveness of educational programs in schools and, as such supporting multicultural students who encounter difficulties related to school experiences in South Korea.

Before delving into the theoretical backgrounds, it is important to define “multicultural families,” “multicultural students,” and “multicultural family support services” as terms used in this research. As per South Korea's Multicultural Family Support Act, Article 2, a “multicultural family” is one that includes marriage between immigrants and those born with Korean nationality, as well as families with members who have been naturalized. “Multicultural students” are those hailing from such families. “Multicultural family support services” are provided by the Multicultural Support Centers under the purview of South Korea's Ministry of Gender Equality and Family and aim to help multicultural families and family members acculturate into Korean society, offering various programs, such as language class, psychological counseling, parenting education, educational support, and career guidance.

## Theoretical framework and hypothesis

### Multicultural families and students in South Korea

In contemporary South Korea, households with diverse cultural heritages, termed “damunhwa gajok” are gaining prominence. While the nation traditionally valued ethnic homogeneity, greater diversity is rapidly being introduced to the society due to the globalization and 21st century socio-cultural shifts, including the growing number of international marriages. In a recent study by Statistics Korea (Kim, 2023, 2023a), the number of multicultural households in South Korea was recorded as 397,805 in the 2022 census. This figure represents 0.8% of the total population, a 3.7% increase from 2021 and a 21.1% increase compared to 2017. Of these households, 42.8% were led by naturalized Koreans, and 37.2% by marriage immigrants. The most prevalent nationality within these groups was Korean-Chinese at 32.3%, closely followed by Vietnamese at 21.8% and Chinese at 19.0%.

This rise in multicultural households in South Korea has directly impacted the student demographics, with Kim (2021), noting that there was a notable increase in multicultural students from 46,955 in 2012 to 160,056 in 2021. This growth is significant, especially considering the overall decline in the student population during the same period. By 2021, multicultural students constituted 3.0% of the student population, up from just 0.7% in 2012. Delving into the specifics, Vietnamese students represented the largest group among multicultural students, their numbers growing from 26.5% of the multicultural student population in 2017 to 32.2% in 2021, followed by students from China (excluding Korean-Chinese), then the Philippines, and Korean-Chinese students. A notable decline was observed, however, among students of Japanese descent, their proportion decreasing from 10.5% in 2017 to 5.2% in 2021.

In response to the growth rate of multicultural families and students, the government has intensified its support and initiated multicultural education programs for such individuals. Despite these efforts, however, families and students from diverse backgrounds still grapple with many challenges, particularly in the education sector. For instance, Kim (2021) highlighted that, in 2017, dropout rates for multicultural middle and high school students stood at 1.47 and 2.11%, respectively. These figures were significantly higher than the rates for the general student population, which were 0.7% for middle schools and 1.5% for high schools. The predominant factors behind these high dropout rates encompassed non-attendance, conflicts with peers or educators, plans for international studies or school transfers, financial constraints, cultural adaptation struggles, and academic difficulties (Kim, 2021). Such data underscore the pressing academic and social challenges multicultural students encounter. Given this backdrop, it is imperative to assess the effect of the government's initiatives in assisting multicultural students to navigate and surmount their challenges in educational settings.

### Multicultural family support centers

The Multicultural Family Support Centers are dedicated to assisting immigrants by marriage and their families to lead enjoyable and stable lives in South Korea (Kang, 2012), their primary focus being to address the distinct educational challenges these families and children encounter within the country's educational system and Korean culture, specifically, in developing programs that promote family integration and provide specialized educational support tailored to the needs of these families and students. At the heart of their such programs are carefully crafted supporting areas, including family, gender equality, human rights, social integration, counseling, and resource integration. Among these programs, Korean language courses are seen as vital for the marriage immigrants and their children to enhance both ordinary and educational lives. Additionally, the centers offer services dedicated to cultural adaptation, ensuring individuals gain a thorough understanding of, and are able to adapt to, Korean societal norms and practices.

As of 2023, 211 multicultural family support centers have been established to offer free services to marriage immigrants and their families (Kim, 2023b). For example, membership of the Danuri Multicultural Family Support Web Portal (www.liveinkorea.kr) is available to marriage immigrants and their families who can freely enroll in programs through the Danuri helpline or by registering online on the Danuri webpage. Additionally, individuals can voluntarily search for a nearby center by looking up their region on the Danuri webpage. The centers operate with the support of government funding and private sector sponsorships, and are managed by various organizations, including regional or private institutions such as the global center. Additionally, Ansan-si in Gyeonggi-do, an area with a high population of foreigners, has its own autonomous foreign residents' center, providing administrative services.

These support centers generally provide a variety of services that encompass group education on numerous social issues, Korean language learning, community outreach programs, counseling, and information services. Additionally, their support broadens to include a wide spectrum of educational needs, addressing more than just language and cultural aspects. These include offering counseling services to address a range of challenges, such as academic guidance for school admissions, support for daily school and home lives, and psychological counseling with professionals. Moreover, the centers also advocate early social and cultural education for immigrating students and cater to specialized educational needs, providing inclusive academic support. With a clear understanding of the pivotal role families play in shaping educational trajectories of students, the centers actively encourage parental involvement. This underscores their commitment to seamlessly integrating multicultural families into the tapestry of South Korean society.

The Korean Institute for Healthy Family (2022) reported that the multicultural family support centers offer a variety of programs across five key areas: family support, gender equality, human rights, social integration, and counseling. These include both mandatory and elective programs tailored to each area's specific needs. Overall, 1,525,520 individuals have participated in these programs at 230 multicultural family support centers, the family support program showing the highest participation with 767,389 individuals, followed by the social integration program with 445,581, the human rights program with 193,569, the counseling program with 66,924, and the gender equality program with 52,057 individuals. However, despite the government's efforts to reform the multicultural family act and support programs, the fact remains that there are fewer multicultural students who pursue further studies or enter higher education (Son, 2023). Additionally, the annual report from the Korean Institute for Healthy Family tracks program participation but does not include data on student involvement from different school levels or assess the effectiveness of these programs. Therefore, it is essential to assess the effectiveness of the government's plans and programs in supporting multicultural students and such analysis may provide insights for potential reforms.

### Theoretical approach

Adolescence is a crucial phase marked by individuals delving into their evolving identities and understanding their place in society (Steinberg and Morris, 2001). As they undergo physical, psychological, and social maturation, they encounter rapid developmental changes and challenges, and are more vulnerable to problematic behaviors than in earlier stages. Cultural shifts and acculturation during adolescence also significantly influence one's quality of life in the long term.

In this section, we analyze literature on how government initiatives affect multicultural students, with a focus on acculturative stress, peer relations, and academic adjustment. We review research to understand how these studies address key variables and assess the effectiveness of government programs. Moreover, we prioritize longitudinal studies on multicultural adolescents to obtain a comprehensive picture of how such l students in South Korea exhibit unique characteristics in each key variable.

According to previous empirical studies, the trajectories of acculturative stress, peer relations, and school adjustment vary among students with multicultural backgrounds. Acculturative stress has shown inconsistencies over the years (e.g., Phinney et al., 2001; Ying, 2005; Tartakovsky, 2007; Bong et al., 2018), whereas, in contrast, peer relations have displayed an increase during adolescence (Kim et al., 2012; No et al., 2016; Son et al., 2017). However, unlike the general population, which exhibits a V-type trajectory (Kim, 2008, 2016; Lee and Cho, 2010), the level of school adjustment continues to decrease for students with multicultural backgrounds (Kim et al., 2012; Park and Oh, 2014; No et al., 2016; Son et al., 2017). The developmental details of the key variables are discussed below.

#### Acculturative stress

Acculturation refers to the adaptations made by both groups and individuals when exposed to different cultures. At the group level, acculturation leads to socioeconomic shifts (Redfield et al., 1936), while, at the individual level, it results in behavioral and value changes (Graves, 1967). Cross-cultural psychologists aim to connect both these levels, seeking to link individual changes to the broader cultural context and to understand the variability in acculturative outcomes (Berry et al., 1986). Within this framework, *acculturative stress* describes the range of psychological responses observed among individuals, expanding the concept of cultural shock to include both psychological distress and maladaptive behavior (Williams and Berry, 1991; Berry, 2006). Research has shown that five key elements influence acculturative stress, including the mode and phase of acculturation, the larger societal context, and the characteristics of both acculturating group and individual (see Berry and Kim, 1988 for a review). In the context of acculturation, Berry (2006) elucidated four distinct strategies, namely assimilation, separation, integration, and marginalization, and highlighted two fundamental challenges individuals face during the process of cultural adaptation: maintaining one's indigenous cultural identify and achieving a level of integration with other cultural groups (see Berry, 2006 for a review). Among these factors, social support may emerge as a key aspect, influencing the needs of both the group and the individual during the acculturation process.

A large body of research on multicultural adolescents has unveiled diverse trajectories of acculturative stress which, instead of conforming to a single developmental pattern, exhibit a variety of patterns. For instance, some studies highlight a U-shaped trajectory, where acculturative stress initially decreases and then resurges (Phinney et al., 2001). In contrast, other research outlines a trajectory in which acculturative stress peaks initially and then diminishes (Tartakovsky, 2007). Another set of findings suggests a consistent and linear reduction in acculturative stress over time (Ying, 2005; Bong et al., 2018). These varied results emphasize the complexities of the cultural adaptation process in the multicultural adolescent population, highlighting the need for a subtle understanding of the subject.

#### Peer relations

Peer relations refer to the acceptance and support individuals receive from those in similar developmental stages or age groups through interaction. It represents one of the most significant social relationships for adolescents, its importance emerging during adolescence due to a growing need for independence from parents and the increased time spent with peers during this developmental stage. Positive peer relations correlate with positive psychological and societal outcomes for adolescents; for instance, in promoting positive school adjustment (Wentzel et al., 2014), buffering stress levels through emotional support from peers, and significantly influencing adjustment (Pryor-Brown and Cowen, 1989; Conrad and Hammen, 1993). They are also associated with high self-esteem (Keefe and Berndt, 1996).

In the context of South Korea, adolescents with multicultural backgrounds encounter numerous challenges, including acculturation stress, micro-discrimination, academic problems, and more (Ministry of Gender Equality and Family, 2021). Empirical studies focusing on these groups suggest that peer relations serve a beneficial role for them, such as moderating the relationship between acculturative stress and depression: maintaining strong peer relationships promotes positive school adjustment (Hong and Ahn, 2021). Moreover, support from peers can moderate the negative effect of acculturative stress on social withdrawal for those groups (Mo, 2018). In sum, peer relations play a pivotal role during adolescence (Kim et al., 2012; No et al., 2016; Son et al., 2017), protecting multicultural students from negative situations and enhancing their performance, especially within the school environment. This suggests its potential as a significant metric in assessing government policies concerning these students.

#### School adjustment

School adjustment refers to the adoption of positive social behaviors that lead to both individual development and social integration. Such behaviors not only ensure harmonious group interactions but also result in gaining social approval and acceptance. On an individual level, these behaviors manifest as achieving personal skills, a sense of autonomy, and emotional and social wellbeing (Wentzel, 2013). Hence, school adjustment may be linked to behavioral issues during adolescence, encompassing relationships with peers and teachers, engagement in learning activities, and adherence to school values (Bae, 2022). Yet, more intriguingly, adjustment to school in South Korean educational environments appears to vary based on the grade level of the students. The education system in South Korea follows a 6-3-3 structure within a 12-year framework: 6 years of elementary school for ages 6–12 (Grades 1–6), 3 years of middle school for ages 13–15 (Grades 7–9), and 3 years of high school for ages 16–18 (grades 10–12). Predominantly, empirical studies reveal a discernible decline in school adjustment as students transition from the latter stages of elementary school to middle school (Lee and Cho, 2010). This trajectory, interestingly, demonstrates an inversion with an observed increase in adjustment from middle school to high school (Kim, 2008; Jwa, 2012; Bae, 2022).

However, students with different backgrounds and at various school levels experience unique patterns of development in their school adjustment (Park and Oh, 2014; Lee and Chung, 2016). Lee and Chung (2016), for instance, found that the school adjustment level decreases as the grade level increases for children from vulnerable groups. Nevertheless, for children from multicultural families, there is a decline in average school life adjustment as they progress from the lower grades of elementary school to the upper grades and then to middle school (Park and Oh, 2014). Thus, to encapsulate these research findings, school adjustment can vary significantly based on grade level and the background of the students. Moreover, these findings can serve as crucial indicators for formulating government policies and assessing their effects on adolescents.

### Hypothesis

Evidence suggests that, during early adolescence and school transitions, there would be a decrease in changes related to acculturative stress (Phinney et al., 2001; Ying, 2005; Bong et al., 2018) and school adjustment (Park and Oh, 2014; Lee and Chung, 2016). However, there is a prevailing opinion that peer relations perceived by students from multicultural families hardly show a certain marked changing pattern (Kim et al., 2012; No et al., 2016; Son et al., 2017). Thus, we hypothesized that multicultural students' perceptions in acculturative stress and school adjustment would follow decreasing developmental trajectories over time. With respect to students' perception of peer relations, our objective was to identify its developmental pattern without making a specific assumption. We also hypothesized that multicultural family support services would have both immediate and delayed impacts on the longitudinal changes in acculturative stress, peer relations, and school adjustment.

## Method

### Participants and procedure

We used data from the Multicultural Adolescents Panel Survey (MAPS) 2011 for this study. MAPS 2011 is an ongoing longitudinal follow-up study focusing on the multicultural youth and policy development, and is annually collected by the National Youth Policy Institute in South Korea. The survey data from MAPS 2011 was designed to start in 2011, with participants who were 4th graders in elementary school (approximately 9–10 years old), and is projected to end in 2025, when participants will turn 24 years old. The MAPS data is collected using a stratified, multistage cluster design to obtain a sample representative of multicultural families, and its questionnaire is administered in Korean.

In this study, we used only four of the 10 waves of multicultural data: wave 3, collected in 2013 when the students were in the sixth grade; wave 4, collected in 2014 when the students were transitioning to junior high school; wave 5, collected in 2015 when the students were in the eighth grade; and wave 6, collected in 2016 when most students had graduated from junior high schools. A total of 1,635 students participated in the survey in 2013. However, prior to data analysis, we excluded 192 students who did not respond to any survey items from 2013 to 2016. Eventually, we included responses of 1,443 students (706 boys, 737 girls) from various cities, including rural, suburban, and urban areas, in the final analysis.

### Measure

#### Utilization of multicultural family support services

The utilization of multicultural family support services was assessed with a single question from 2013 (Wave 3) to 2016 (Wave 6). Students were asked whether they had experience using multicultural family support services within a year. The response was coded dichotomously as 1 for yes and 0 for no.

#### Acculturative stress

We adopted four items from MAPS (from Wave 4 to Wave 6) to measure acculturative stress resulting from various sources such as social, attitudinal, familial, and environmental contexts as well as perceived discrimination (majority group stereotypes) toward migrant populations. The four items include, “I feel stressed living in Korea,” “I do not want to go to school because my parents are not from Korea,” “I am stressed because I am not good at Korean,” and “People around me stress me out to act like Koreans.” Each item was rated on a 4-point Likert-type scale ranging from *1 (not at all)* to *4 (very much so)*. Higher scores indicate stronger agreement with the statement. The Cronbach alpha coefficients were 0.87, 0.87, and 0.85 for 2014 (wave 4), 2015 (wave 5), and 2016 (wave 6), respectively.

#### Peer relations

We used two items (i.e., “I fit in well with the peers in my class”; “The peers in my class respect what I say when playing or participating in group activities”) from MAPS (from wave 4 to wave 6). Each item was rated on a 4-point Likert-type scale ranging from *1 (not at all)* to *4 (very much so)*. Higher scores indicate stronger agreement with the statement. The Cronbach alpha coefficients were 0.72, 0.68, and 0.68 for 2014 (wave 4), 2015 (wave 5), and 2016 (wave 6), respectively.

#### School adjustment

We used four items from MAPS (from wave 4 to wave 6) to measure motives, attitudes, and practices of students related to their adjustment to school (e.g., “I enjoy school classes”; “I always do my school assignments”). Each item was rated on a 4-point Likert-type scale ranging from *1 (not at all)* to *4 (very much so)*. Higher scores indicate stronger agreement with the statement. The Cronbach alpha coefficients were 0.80, 0.80, and 0.79 for 2014 (wave 4), 2015 (wave 5), and 2016 (wave 6), respectively.

### Study design and analysis

**Figure 1:**
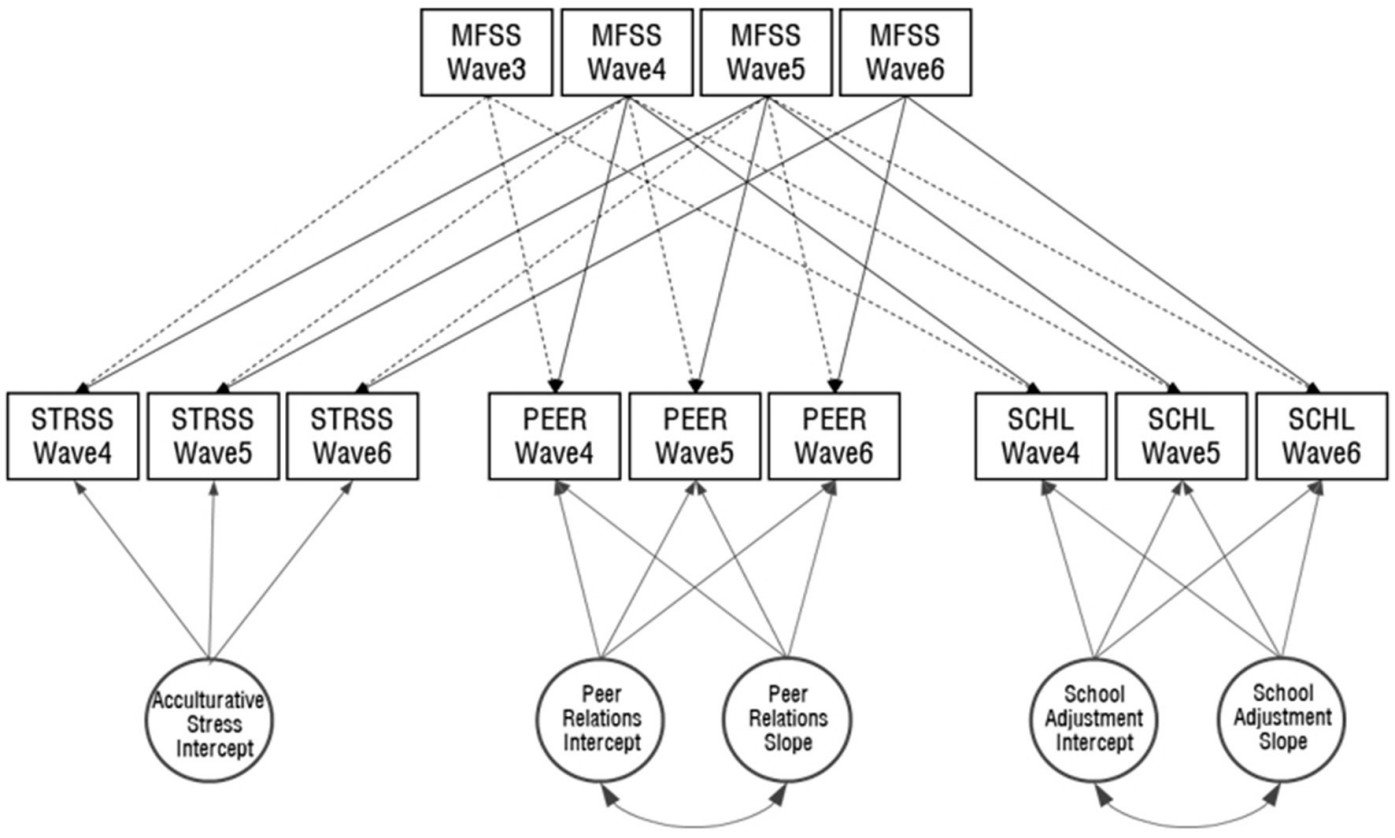
Time–concurrent and time–lagged effects model. The dashed lines show the delayed effect, while the solid lines represent the concurrent effect, of multicultural family support services. MFSS, multicultural family support services; STRSS, acculturative stress; PEER, peer relations; SCHL, school adjustment.

Time-concurrent and time-lagged models, as well as latent growth curve models (Duncan et al., 2013), were implemented using SPSS 23.0 and Amos 23.0. Given the theories being tested (see Figure 1), the time-concurrent and time-lagged models examine whether the utilization of multicultural family support service at an earlier time point (*t*−1) is predictive of acculturative stress, peer relations, and school adjustment at a later time point (*t*). At the same time, the models examine whether the utilization of multicultural family support services at a certain time (*t*) is predictive of acculturative stress, peer relations, and school adjustment at the same time point (*t*). The latent growth curve model examined the developmental growth trajectories of acculturative stress, peer relations, and school adjustment over three time points (waves 4–6).

## Results

### Descriptive statistics and correlations

Table 1 presents the correlations, means, standard deviations, skewness, and kurtosis for the study variables. The mean scores of each measured variables at wave 4–6 ranged from 1.21 to 1.26 for acculturative stress, 3.16–3.19 for peer relations, and 2.90–2.92 for school adjustment, respectively. The skewness and kurtosis of the three variables throughout the three waves remained between −0.51 and 2.12, and between 0.18 and 4.63, respectively. As their absolute values were below 3.0 and 10.0, the data were assumed to follow the normal distribution (Kline, 2010). The bivariate correlation test among utilization of multicultural family support services, acculturative stress, peer relations, and school adjustment revealed significant weak to moderate correlations between the variables across time (ranged from −0.28 to 0.48, *p*s < 0.001), except for the correlations between multicultural family support services and peer relations.

**Table 1:** Descriptive statistics and correlation coefficients among variables.

**Variable**		**1**	**2**	**3**	**4**	**5**	**6**	**7**	**8**	**9**	**10**	**11**	**12**	**13**
1	Multicultural family support services, wave 3	–												
2	Multicultural family support services, wave 4	0.15	–											
3	Multicultural family support services, wave 5	0.17	0.29	–										
4	Multicultural family support services, wave 6	0.15	0.25	0.34	–									
5	Acculturative stress, wave 4	−0.06	0.02	0.01	0.00	–								
6	Acculturative stress, wave 5	0.01	−0.02	−0.07	−0.04	0.31	–							
7	Acculturative stress, wave 6	−0.04	−0.05	−0.06	−0.04	0.29	0.32	–						
8	Peer relations, wave 4	0.04	0.04	0.01	0.00	−0.28	−0.21	−0.16	–					
9	Peer relations, wave 5	0.04	0.02	0.01	0.03	−0.23	−0.27	−0.24	0.48	–				
10	Peer relations, wave 6	−0.00	0.03	0.02	0.05	−0.19	−0.20	−0.26	0.44	0.57	–			
11	School adjustment, wave 4	0.04	0.05	0.00	0.00	−0.24	−0.16	−0.16	0.51	0.37	0.34	–		
12	School adjustment, wave 5	0.00	0.01	0.06	0.02	−0.14	−0.19	−0.18	0.31	0.48	0.36	0.54	–	
13	School adjustment, wave 6	0.02	0.05	0.03	0.09	−0.19	−0.17	−0.19	0.32	0.42	0.51	0.52	0.59	–
	*M*	0.55	0.32	0.31	0.25	1.21	1.26	1.22	3.19	3.19	3.16	2.92	2.90	2.90
	SD	0.50	0.47	0.46	0.43	0.40	0.44	0.39	0.55	0.51	0.49	0.56	0.56	0.54
	Cronbach's alpha (α)	–	–	–	–	0.87	0.87	0.85	0.72	0.68	0.68	0.80	0.80	0.79
	Number of items used in the final analysis	1	1	1	1	4	4	4	2	2	2	4	4	4
	Skewness	−0.20	0.79	0.82	1.18	2.12	1.97	1.88	−0.51	−0.36	−0.31	−0.04	−0.07	−0.18
	Kurtosis	−1.96	−1.38	−1.33	−0.62	4.63	4.15	3.77	1.24	0.97	1.28	0.18	0.23	0.69

Correlation coefficients >0.06 in absolute value are significant at the 0.05 level. Multicultural family support services are coded no = 0 and yes = 1. Response scales ranged between 1 and 4 for the acculturative stress, peer relations, and school adjustment.

### Model specification

We performed latent growth modeling based on the following two-step approach (Bollen and Curran, 2006). In the first step, we tested the unconditional latent growth model (see Figure 1) to examine intra-individual differences in the developmental growth trajectories of acculturative stress, peer relations, and school adjustment across three time points (wave 4–6). In our next step, we constructed a conditional model to examine the time-concurrent and time-lagged effects of multicultural family support services variable on the three dependent variables—acculturative stress, peer relations, and school adjustment. Model fit was deemed satisfactory when the comparative fit index (CFI) and Tucker-Lewis index (TLI) values were above 0.90 and the root mean square error of approximation (RMSEA) was close to 0.06 (Browne and Cudeck, 1992; Hu and Bentler, 1999).

#### Unconditional latent growth model

We tested the unconditional LGM for our longitudinal dependent variables based on the model fit indexes and chi-square differences of default model (no-growth) and the lineal growth model of each variable. Table 2 shows the fitting indexes and chi-square differences of each tested models. The fit indexes showed that the growth model fit the data well for peer relations and school adjustment. For acculturative stress variable, however, the fit indexes as well as the chi-square difference test supported the default model (no growth). As reported in Table 3,^1^ the initial level of peer relations was 3.196 (*p* < 0.001), and it decreased significantly over the three measurement points (slope = −0.017, *p* < 0.05). The variances of intercept (σ^2^ = 0.151, *p* < 0.001) and slope (σ^2^ = 0.021, *p* < 0.001) were also significant, indicating that both initial levels and changes of peer relations significantly differed between individuals. The intercept and slope were significantly negatively correlated (*r* = −0.016, *p* < 0.05), indicating that higher initial values of peer relations were related to steeper declines across time. As to school adjustment, at the mean level, the intercept was found to be significant (*p* < 0.001) whereas the slope did not reach the significance level. The variances of intercept (σ^2^ = 0.182, *p* < 0.001) and slope (σ^2^ = 0.017, *p* < 0.001), indicating that inter-individual initial levels and changes of school adjustment significantly differed. The correlation between the intercept and slope was not significant.

**Table 2:** Fitting indexes of unconditional latent growth model of each variable.

**Model**	**Δχ^2^ (Δ*df*)**	**CFI**	**TLI**	**RMSEA**
**Acculturative stress**				
Default model	6.387 (3)	0.956	0.933	0.050
Growth model		0.966	0.797	0.087
**Peer relations**				
Default model	17.736^***^ (3)	0.983	0.974	0.053
Growth model		0.999	0.991	0.031
**School adjustment**				
Default model	9.638^*^ (3)	0.995	0.992	0.032
Growth model		1.000	1.003	0.000

^*^p < 0.05.

^***^p < 0.001.

**Table 3:** Estimated parameters of each latent growth model.

**Model**	**Mean**		**Variance**		**I_Corr_S**
	**Intercept**	**Slope**	**Intercept**	**Slope**	
Acculturative stress	1.228^***^	–	0.051^***^	–	–
Peer relations	3.196^***^	−0.017^*^	0.151^***^	0.021^***^	−0.016^*^
School adjustment	2.918^***^	−0.010	0.182^***^	0.017^***^	−0.013

^*^p < 0.05.

^***^p < 0.001.

#### Conditional latent growth model: time-concurrent and time-lagged effects

**Figure 2:**
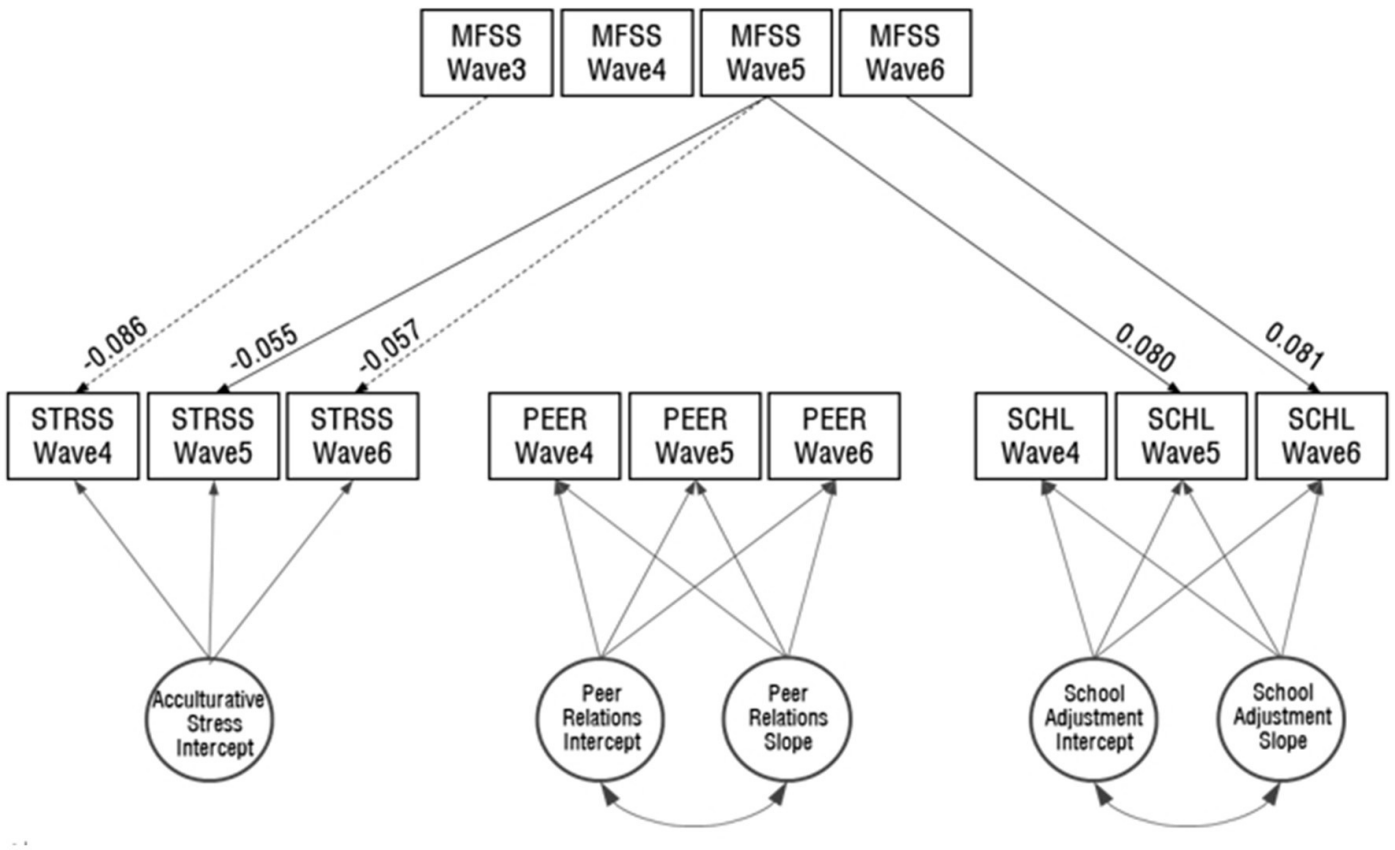
Estimates for time–concurrent and time–lagged effects of multicultural family support services on acculturative stress, peer relations, and school adjustment. Only statistically significant predicted paths for both delayed and concurrent effects are indicated at *p* < 0.05. The dashed lines show the delayed effect, while the solid lines represent the concurrent effect, of multicultural family support services. Coefficients of variance and error variances have been omitted to maintain graphical clarity. MFSS, multicultural family support services; STRSS, acculturative stress; PEER, peer relations; SCHL, school adjustment.

Based on the tests of unconditional LGM, we proceeded to our next step to examine the conditional latent growth model by assuming linear growth for peer relations and school adjustment variables and no significant growth for acculturative stress over three time points. As presented in Figure 1, our research model includes the predictive paths from multicultural family support services at wave 3–6 respectively to the three dependent variables at wave 4–6. The results showed satisfactory fits, χ^2^(43) = 198.780, *p* < 0.001, CFI = 0.96, TLI = 0.915, RMSEA = 0.05, indicating that our research model fit the data well. As reported in Table 4 and Figure 2, multicultural family support services (MFSS) showed statistically significant delayed and concurrent effects on acculturative stress. MFSS at wave 3 and 5 negative predicted acculturative stress at wave 4 (β = −0.07, *p* < 0.001) and 6 (β = −0.05, *p* < 0.05), respectively. MFSS at wave 5 also significantly negatively predicted acculturative stress at the same time point (β = −0.05, *p* < 0.05), indicating that more multicultural family support services experienced by individuals tended to be related to lower levels of acculturative stress at the same and/or later time points. While the delayed effects of MFSS on school adjustment only reached marginally significant statistical level from MFSS at wave 3 (*p* = 0.056), its concurrent effects of wave 5 and 6 were found to be significant (βs = 0.10, *p*s < 0.001). That is, more multicultural family support services experienced by individuals tended to be related to the higher levels of school adjustment at the same time points. Neither delayed nor concurrent effects of MFSS on peer relations reached statistically significant levels.

**Table 4:** Estimates for time–concurrent and time–lagged effects of multicultural family support services on acculturative stress, peer relations, and school adjustment.

**Paths**	***B***	**β**	**SE**	***p*-value**
**Delayed effects**				
MFSS, wave 3 → acculturative stress, wave 4	−0.069	−0.086	0.016	<0.001
MFSS, wave 3 → peer relations, wave 4	0.029	0.026	0.023	0.214
MFSS, wave 3 → school adjustment, wave 4	0.043	0.038	0.023	0.056
MFSS, wave 4 → acculturative stress, wave 5	0.031	0.033	0.024	0.188
MFSS, wave 4 → Peer relations, wave 5	0.017	0.016	0.024	0.482
MFSS, wave 4 → school adjustment, wave 5	−0.034	−0.028	0.026	0.200
MFSS, wave 5 → acculturative stress, wave 6	−0.049	−0.057	0.022	0.028
MFSS, wave 5 → peer relations, wave 6	0.017	0.016	0.027	0.535
MFSS, wave 5 → school adjustment, wave 6	0.010	0.008	0.029	0.731
**Concurrent effects**				
MFSS, wave 4 → acculturative stress, wave 4	0.021	0.024	0.021	0.326
MFSS, wave 4 → peer relations, wave 4	0.021	0.018	0.029	0.460
MFSS, wave 4 → school adjustment, wave 4	0.034	0.028	0.028	0.231
MFSS, wave 5 → acculturative stress, wave 5	−0.052	−0.055	0.024	0.032
MFSS, wave 5 → peer relations, wave 5	0.038	0.035	0.025	0.129
MFSS, wave 5 → school adjustment, wave 5	0.097	0.080	0.027	<0.001
MFSS, wave 6 → acculturative stress, wave 6	−0.016	−0.017	0.024	0.501
MFSS, wave 6 → peer relations, wave 6	0.038	0.033	0.026	0.148
MFSS, wave 6 → school adjustment, wave 6	0.100	0.081	0.027	<0.001

MFSS, multicultural family support services.

## Discussion

This study investigated the longitudinal trajectories of acculturative stress, peer relations, and school adjustment among adolescents (from 6th to 9th graders) with multicultural backgrounds in South Korea. The findings revealed that peer relations and school adjustment perceived by adolescents demonstrated linear changes declining across 3-year time points while the trajectories of acculturative stress exhibited no significant changes during the same period. The non-significant developmental changes in acculturative stress are seen linked to the floor effects resulting from its low mean level. However, considering that the initial level and developmental trajectories of acculturative stress also significantly differed between individuals, it deserves our attention to find major influencing factors of acculturative stress as well as peer relations and school adjustment.

We focused on examining the effects of the utilization of multicultural family support services (MFSS) on acculturative stress, peer relations, and school adjustment. Particularly, our focus was on how MFSS provided at an earlier time point predict adolescents' perceptions of acculturative stress, peer relations, and school adjustment, both at the same time point and later. The findings partially support the time-concurrent and time-lagged effects of MFSS on the dependent variables. First, using MFSS in wave 3 and 4 (5th and 6th Grade) significantly reduced the levels of acculturative stress in the subsequent year (6th and 7th Grade). Using the support services in wave 5 (7th Grade) also significantly reduced acculturative stress at the same time point. Similarly, the utilization of MFSS in wave 5 and 6 (7th and 8th Grade) showed positive predictions to school adjustment in those respective time points. As to peer relations, however, there were no concurrent or delayed influences across any time points.

School adjustment perceived by adolescents with multicultural backgrounds showed a decreasing pattern over time in the current study, but the change was not large enough to reach statistical significance. These results are consistent with previous findings reporting temporal stability of school adjustment perceived by adolescents from multicultural families (e.g., Kim and Jung, 2020; Song and Shin, 2020). In addition, contrary to previous research, we found that acculturative stress did not show developmental change over time, even though students exhibited significant differences in their initial levels (intercepts). These findings may suggest that the acculturative stress perceived by multicultural students may be buffered by both the concurrent and delayed impacts of MFSS. However, more importantly, even though the concurrent and delayed impacts of MFSS on acculturative stress are evident at a few time points, they do not significantly influence the developmental trajectories of school adjustment and acculturative stress across all time points. Furthermore, when considering the influence of MFSS over time, it becomes evident that school adjustment is primarily linked with MFSS during Grades 8 and 9, while acculturative stress experienced by multicultural students is most profoundly impacted by MFSS during their transition from elementary to middle school, specifically in Grade 7. Therefore, future research should pay more attention to the developmental trajectories of school support and acculturative stress in multicultural students and further investigate the factors influencing their developmental changes. Moreover, there is a need for a meticulous examination of the effects of MFSS during these students' pivotal transition in Grades 7 and 9, as well as the types of programs they opt for. Furthermore, given the intermittent impact of MFSS across time, it suggests that policy makers should develop tailored programs based on students' environments and grade levels, complemented by needs assessments.

Moreover, we found that peer relations among adolescents from multicultural backgrounds decrease from late elementary school through 9th Grade. This is in contrast to findings from previous research highlighting temporal stability of peer relations (Kim et al., 2012; No et al., 2016; Son et al., 2017). In other words, the current findings show that adolescents feel less supported and accepted by their peers as the grade level increases. These results can be understood in the context of Korean society and the significance of peer relations might be heightened due to communication difficulties between parents of multicultural families and their children caused by language barriers (Oh, 2015). In fact, many multicultural families communicate in Korean rather than the native language of the foreign parent, and only 27.3% of multicultural students reported having the motivation to master their foreign parent's native language (Ministry of Gender Equality and Family, 2021). In contrast to the elevated need for support from their peers, however, adolescents with multicultural backgrounds in South Korea exhibited a trajectory of gradual social withdrawal (Jung and Lee, 2021), implying that they might experience more difficulties in interpersonal relationships. Further research is needed to investigate the factors influencing multicultural students' peer relations and to develop effective intervention programs to promote such relations.

Furthermore, the effects of MFSS on peer relations were found to be nonsignificant in the current study. As such, the fact that the existing MFSS did not play a meaningful role in buffering the decreases in peer relations requires attention. Indeed, much effort has been expended to help integrate multicultural families into the Korean society by providing them with MFSS following the Multicultural Families Support Act in 2012 and 2016 (Ministry of Government Legislation, 2016). However, numerous studies have demonstrated concerns about the effects of MFSS (Hwang, 2016). Specifically, findings have shown that the current MFSS do not fully reflect the differences among diverse ethnic groups. For example, in research to examine the effects of MFSS on participants' life satisfaction, utilizing the service had positive effects only for Chinese among immigrants with various ethnic backgrounds, such as Filipinos, Vietnamese, and Korean Chinese (Sung et al., 2013). Similarly, while the current MFSS programs have centered on integrating the immigrants, particularly wives, into society, there needs to be a variety programs designed to support various members of multicultural families and of various age groups, such as second and third generations (Kang, 2012). The current programs for children and adolescents are confined to help Korean language acquisition and academic improvement with only a few revolving around school adjustment and prevention of alienation. Considering the diversity of the characteristics of multicultural families and the issues that members from multicultural families have, MFSS should include diverse programs that deal with specific and authentic solutions. Systematic program changes are also needed as the focal issues of multicultural families change over time depending on the extent to which they are integrated into society.

While this research enhances our understanding of the dynamic patterns of acculturative stress, peer relations, and school adjustment among adolescents with multicultural backgrounds, it does come with several limitations. For instance, the current findings are based on the characteristics of multicultural students living in South Korea. Additionally, this study focused solely on the responses of MFSS participants to determine the impact on the diverse patterns of multicultural adolescent development. To further our knowledge of how government programs can support multicultural adolescent development, future research should consider the following two-track investigations. First, it is necessary to explore the roles of various individual and contextual assets; for example, significant individual differences exist in the extent of changes in acculturative stress, peer relations, and school adjustment over time. By identifying which assets positively impact adolescent development and understanding their effects on the programs, we can better determine where to focus and how to tailor programs for multicultural adolescents. As such, future longitudinal studies should factor in both personal and contextual assets to comprehensively analyze the diverse developmental trajectories of multicultural adolescents. Second, while so doing, we should examine whether specific programs or support mechanisms of MFSS play a role in the development of multicultural students in South Korea. For example, Lee and Lee (2013) examined the effectiveness of the program designed to improve peer relations of children from multicultural families and demonstrated that it had positive effects on the number of friends and the perceptions of persistency of the peer relations.

Despite these challenges, there are several important implications to consider. First, developing meaningful relationships with peers is crucial for students with multicultural backgrounds in order to adjust to Korean society. Teachers and practitioners should recognize the significance of peer interactions when creating or implementing programs for these students. Additionally, since government programs like MFSS have limited long-term sustainability in terms of their impact on acculturative stress, peer relations, and school adjustments, practitioners and policy makers must take these results into account when formulating prevention strategies. Lastly, prevailing perceptions of developmental trajectories among multicultural adolescents should be reevaluated to design more nuanced and effective school programs. Our research aims to enhance our understanding and promotion of the varied patterns of change and program effects that typify multicultural adolescents.

## Data availability statement

Publicly available datasets were analyzed in this study. This data can be found at: https://www.nypi.re.kr/archive/board?menuId=MENU00221.

## Ethics statement

Ethical review and approval was not required for the study on human participants in accordance with the local legislation and institutional requirements. Written informed consent from the patients/participants or patients/participants legal guardian/next of kin was not required to participate in this study in accordance with the national legislation and the institutional requirements.

## Author contributions

HA: Writing—original draft, Writing—review & editing. JL: Writing—original draft, Writing—review & editing. YJ: Writing—original draft, Writing—review & editing.

## Footnotes

^1^When the growth model of acculturative stress was tested, its variances of intercept (σ^2^ = 0.063, *p* < 0.001) and slope (σ^2^ = 0.010, *p* < 0.001) were also found to be significant, indicating that there were significant individual differences in the initial levels and changes of acculturative stress.

## References

[B1] Albeg L. J. Castro-Olivo S. M. (2014). The relationship between mental health, acculturative stress, and academic performance in a Latino middle school sample. Contemp. Sch. Psychol. 18, 178–186. 10.1007/s40688-014-0010-1

[B2] Bae S. M. (2019). The relationship between bicultural identity, acculturative stress, and psychological wellbeing in multicultural adolescents: verification using multivariate latent growth modelling. Stress Health 36, 51–58. 10.1002/smi.2912 31736213

[B3] Bae S. M. (2022). The relationship between parental neglect, school adjustment, and smartphone dependence in Korean adolescents: verification using multivariate latent growth modeling. Child Psychiatry Hum. Dev. 10.1007/s10578-022-01485-7 . [Epub ahead of print]. 36576639

[B4] Berry J. W. (2006). “Acculturative stress,” in Handbook of Multicultural Perspectives on Stress and Coping, eds P. T. P. Wong, and L. C. J. Wong (Berlin: Spring Publications), 287–298. 10.1007/0-387-26238-5_12

[B5] Berry J. W. Kim U. (1988). “Acculturation and mental health,” in Health and Cross-Cultural Psychology: Towards Application, eds P. Dasen, J. W. Berry, and N. Sartorius (London: Sage Publications, Inc), 207–236.

[B6] Berry J. W. Trimble J. Olmeda E. (1986). “The assessment of acculturation,” in Field Methods in Cross-Cultural Research, eds W. J. Lonner, and J. W. Berry (London: Sage Publications, Inc), 291–324.

[B7] Bollen K. A. Curran P. J. (2006). Latent curve *Models: A Structural Equation Perspective*. Hoboken, NJ: Wiley-Interscience. 10.1002/0471746096

[B8] Bong C. Jeon Y. Hong S. (2018). A longitudinal study on acculturative stress, social support, and self-esteem within multicultural families: an application of the actor-partner interdependence model using a latent growth model. Stud. Korean Youth 29, 41–69. 10.14816/sky.2018.29.3.41

[B9] Browne M. W. Cudeck R. (1992). Alternative ways of assessing model fit. Sociol. Methods Res. 21, 230–258. 10.1177/0049124192021002005

[B10] Choi M. (1997). Korean students in australian universities: intercultural issues. High. Educ. Res. Dev. 16, 263–282. 10.1080/0729436970160302

[B11] Conrad M. Hammen C. (1993). Protective and resource factors in high and low-risk children: a comparison of children with unipolar, bipolar, medically ill, and normal mothers. Dev. Psychopathol. 5, 593–607. 10.1017/S0954579400006180

[B12] Crockett L. J. Iturbide M. I. Torres Stone R. A. McGinley M. Raffaelli M. Carlo G.. (2007). Acculturative stress, social support, and coping: relations to psychological adjustment among Mexican American college students. Cult. Divers. Ethn. Minor. Psychol. 13, 347–355. 10.1037/1099-9809.13.4.347 17967103

[B13] Duncan T. E. Duncan S. C. Strycker L. A. (2013). An Introduction to Latent Variable Growth Curve Modeling: Concepts, Issues, and Application, 2nd ed. London: Routledge. 10.4324/9780203879962

[B14] Graves T. (1967). Psychological acculturation in a tri-ethnic community. Southwest. J. Anthropol. 23, 337–350. 10.1086/soutjanth.23.4.3629450

[B15] Hong H. Ahn D. (2021). Exploring multicultural families' background characteristics, parental support, peer relationship, school adjustment, and life satisfaction of adolescents. Korean Assoc. Int. Cult. Exch. 10, 111–139. 10.30974/kaice.2021.10.2.5

[B16] Hu L. Bentler P. M. (1999). Cutoff criteria for fit indexes in covariance structure analysis: conventional criteria versus new alternatives. Struct. Equ. Modeling 6, 1–55. 10.1080/10705519909540118

[B17] Hwang M. C. (2016). The effect of multicultural family support service: examining integration with immigrant wives in South Korea. J. Soc. Serv. Res. 42, 630–650. 10.1080/01488376.2016.1216917

[B18] Jung J. Lee D. (2021). The developmental trajectories of social withdrawal among multicultural youth in Korea: Identifying latent classes and testing the impact of both neglectful parenting and supportive friendships. Stud. Korean Youth 32, 57–85. 10.14816/sky.2021.32.1.57

[B19] Jwa H.-s. (2012). The developmental trajectories and factors of adolescents' school adjustment. Korean J. Youth Stud. 19, 1–28.

[B20] Kang B. C. (2012). The status of multicultural family policies and services in Korea: centering around multicultural family support centers. J. Migr. Soc. 5, 143–190. 10.15685/jms.2012.02.5.1.143

[B21] Keefe K. Berndt T. J. (1996). Relations of friendship quality to self-esteem in early adolescence. J. Early Adolesc. 16, 110–129. 10.1177/0272431696016001007

[B22] Kim J.-h. (2023a). Number of multicultural families continues to increase in S. Korea. *Aju Korea Daily*. Available online at: https://www.ajudaily.com/view/20230731132710377 (accessed July 31 2023).

[B23] Kim J.-h. (2023b). Status of Multicultural Family Centers in 2023 [Korean]. Ministry of Gender Equality and Family. Available online at: https://www.mogef.go.kr/mp/pcd/mp_pcd_s001d.do?mid=plc503 (accessed January 31, 2023).

[B24] Kim J. A. (2016). The longitudinal relationship between school adjustment and academic achievement in adolescents on the parenting attitude. Korean J. Counsel. 17, 303–326. 10.15703/kjc.17.2.201604.303

[B25] Kim M. Shin T. Heo Y. (2012). A longitudinal study on the effects of the developmental changes in students' relationship with their teachers and peers on self-determination during middle and high school. Korean J. Educ. Psychol. 26, 429–459. Available online at: https://www.dbpia.co.kr/journal/articleDetail?nodeId=NODE06763894

[B26] Kim N.-y. (2021). Status and Realities of Multicultural Education [2021 Educational issues in Korea, December 2021(1)]. Korean Educational Development Institute. Available online at: https://kess.kedi.re.kr/index (accessed September 18, 2023).

[B27] Kim S.-y. (2023). 2022 Population and Housing Census (Register-based census). Statistics Korea. Available online at: https://kostat.go.kr/board.es?mid=a20107020000andbid=11739andact=viewandlist_no=426675 (accessed September 18, 2023).

[B28] Kim T. K. (2008). A longitudinal study on the changes in school adjustment. J. Future Oriented Youth Soc. 5, 169–188.

[B29] Kim W. Y. Jung N. E. (2020). The effect of mothers' acculturative stress on the self-esteem and the school adjustment of multicultural adolescents. A longitudinal analysis using latent growth modeling. CNU J. Educ. Stud. 41, 257–289. 10.18612/cnujes.2020.41.4.257

[B30] Kline R. B. (2010). Principles and Practice of Structural Equation Modeling, 3rd ed. New York, NY: Guilford Press.

[B31] Korean Institute for Healthy Family (2022). 2022 Family Support Program Annual Report [Korean]. Available online at: https://www.liveinkorea.kr/portal/KOR/board/mlac/boardList.do (accessed December 3, 2023).

[B32] Lee B. C. Song D. Y. (2011). A qualitative study on the school adaptation of multicultural family youth from accompanied entry. Korean J. Soc. Welfare 53, 131–154. 10.20970/kasw.2011.63.4.006

[B33] Lee E. H. Lee K. O. (2013). The effectiveness of peer relation improvement program on peer relation in children of multi-cultural families. J. Korean Acad.-Ind. Coop. Soc. 14, 605–612. 10.5762/KAIS.2013.14.2.605

[B34] Lee H.-j. (2022). Gov't strengthen support for children from multicultural backgrounds. *The Korea Times*. Available online at: https://www.koreatimes.co.kr/www/nation/2023/08/113_323312.html (accessed February 6, 2023).

[B35] Lee H. J. Cho Y. J. (2010). The exploratory study of longitudinal changes and variables predicting school adjustment. Korean J. Youth Stud. 17, 253–278.

[B36] Lee J. Y. Chung I. J. (2016). Predictor variables of developmental trajectories in problem behavior and school adjustment among children from low-income families. Korean Soc. Child Welfare 54, 173–197. Available online at: https://www.dbpia.co.kr/journal/articleDetail?nodeId=NODE07113873

[B37] Lim Y. (2021). Relationship between marriage immigrant mothers' acculturative stress and their adolescent children's career decidedness in South Korea: mediating role s of parenting and school adjustment. Sustainability 13, 14066. 10.3390/su132414066

[B38] Ministry of Gender Equality Family (2021). 2021 Fact-Finding Survey on Multicultural Families. Available online at: http://www.mogef.go.kr/ (accessed September 18, 2023).

[B39] Ministry of Government Legislation (2016). Multicultural Families Support Act of 2012. Available online at: https://www.lawnb.com/Info/ContentView?sid=L000B955DB6C8BC2_0_R10 (accessed September 18, 2023) (in Korean).

[B40] Mo S. H. (2018). Influences of acculturation stress and social support on social withdrawal of multicultural adolescents: moderating effects of teacher and peer-friend factors. Forum Youth Cult. 54, 67–93. 10.17854/ffyc.2018.04.54.67

[B41] No B. Park S. Yi S.-H. Park H. J. (2016). Trajectories of adolescents' peer attachment and their predictors: a multiple group analysis according to gender. Stud. Korean Youth 27, 149–177. 10.14816/sky.2016.27.1.149

[B42] Oh H. J. (2015). A qualitative study on communication difficulty mothers of multicultural families experiences in rearing child. J. Spec. Child. Educ. 17, 215–237. 10.21075/kacsn.2015.17.1.215

[B43] Park H. Oh S. B. (2014). A study of school adaption of multicultural students at primary and secondary school level. Korean Educ. Inq. 32, 35–57. Available online at: https://www.dbpia.co.kr/journal/articleDetail?nodeId=NODE07193168

[B44] Phinney J. S. Horenczyk G. Liebkind K. Vedder P. (2001). Ethnic identity, immigration, and well-being: an interactional perspective. J. Soc. Issues 57, 493–510. 10.1111/0022-4537.00225

[B45] Pryor-Brown L. Cowen E. L. (1989). Stressful life events, support, and children's school adjustment. J. Clin. Child Psychol. 18, 214–220. 10.1207/s15374424jccp1803_3

[B46] Redfield R. Linton R. Herskovits M. J. (1936). Memorandum on the study of acculturation. Am. Anthropol. 38, 149–152. 10.1525/aa.1936.38.1.02a00330

[B47] Son J.-h. (2023). Fewer multicultural students advancing to university. *The Korea Herald*. Available online at: https://www.koreaherald.com/view.php?ud=20230427000785 (accessed April 27, 2023)

[B48] Son S. Lee H. Hong S. (2017). The effects of school learning activities and friendships on adolescents' life satisfaction: a longitudinal study using a piecewise latent growth model. Stud. Korean Youth 28, 57–88. 10.14816/sky.2017.28.3.57

[B49] Song H. G. Shin N. N. (2020). Longitudinal stability and reciprocal effects among adolescents' perceived acculturative stress, self-esteem, and school adjustment in multicultural families. Korean J. Youth Stud. 27, 343–372. 10.21509/KJYS.2020.12.27.12.343

[B50] Steinberg L. Morris A. S. (2001). Adolescent development. Annu. Rev. Psychol. 52, 83–110. 10.1146/annurev.psych.52.1.83 11148300

[B51] Sung M. Chin M. Lee J. Lee S. (2013). Ethnic variations in factors contributing to the life satisfaction of migrant wives in South Korea. Fam. Relat. 62, 226–240. 10.1111/j.1741-3729.2012.00753.x

[B52] Tartakovsky E. (2007). A longitudinal study of acculturative stress and homesickness: high-school adolescents immigrating from Russia and Ukraine to Israel without parents. Soc. Psychiatry Psychiatr. Epidemiol. 42, 485–494. 10.1007/s00127-007-0184-1 17502976

[B53] Wentzel K. Russell S. Baker S. (2014). “Peer relationships and positive adjustment at school,” in Handbook of Positive Psychology in Schools, eds M. J. Furlong, R. Gilman, and E. S. Huebner (London: Routledge), 260–277.

[B54] Wentzel K. R. (2013). “School adjustment,” in Handbook of Psychology, 2nd ed., ed. I. B. Weiner (Hoboken, NJ: John Wiley and Sons), 213–231.

[B55] Williams C. L. Berry J. W. (1991). Primary prevention of acculturative stress among refugees: application of psychological theory and practice. Am. Psychol. 46, 632–641. 10.1037/0003-066X.46.6.632 1952422

[B56] Ying Y. W. (2005). Variation in acculturative stressors over time: a study of Taiwanese students in the United States. Int. J. Intercult. Relat. 29, 59–71. 10.1016/j.ijintrel.2005.04.003

[B57] Yoon L. (2023). Number of Students in Multicultural Families in South Korea 2013-2021. Statista. Available online at: https://www.statista.com/statistics/1249434/south-korea-number-of-students-in-multicultural-families/ (accessed January 20, 2023).

